# Pellagra Not a New Disease in America

**Published:** 1916-09

**Authors:** 


### Pellagra Not a New Disease in America.

The following statements are made by Dr. W. J. W. Kerr of Corsicana, Texas, who, so far as he knows, is the only living surgeon out of sixty-eight who served at the Andersonville prison, and who witnessed something like ten thousand deaths from what he now believes to be pellagra:

"During the year 1864 I was ordered to Andersonville prison, where I found everything in the hospital in a most miserable condition. The prison was built to hold five thousand prisoners, but during the latter part of the summer and early fall we had thirty-six thousand prisoners, and a disease broke out among the prisoners in the stockade that none of the surgeons knew anything about. I had sixty-eight surgeons, and not one of us had ever seen anythink like it.

"I made my report and the C. S. government sent Prof. Joseph Jones there to make post-mortem examinations. He and I performed one hundred and twenty-eight post-mortems, but could not name the disease. No one took it outside the prison who was out a week or such a matter.

"I had nearly two hundred men out on parole, helping to build a hospital, and Captain Wiss and General Winder had nearly or quite as many as I had, and none of them nor any of our own men who guarded there ever took the disease, yet we all ate the same food.

"We lost about ten thousand there from that disease, that now we know was pellagra, but it had a typhoid complication in every case, and but few cases lasted over six or eight weeks after taking. We thought in the beginning that it was a species of scurvy, as there was quite a number of cases of scurvy in the prison, but nearly all was brought there from other prisons. I don't think pyorrhea had anything to do with it. I think the whole cause was due to the crowded and unsanitary condition of the prison, for had it been the diet persons on the outside would have been as liable as those on the inside.

"The guards worked at the top of the wall and were exposed all the time, but they were where they could get the fresh air.

"You ask if I think that the disease had any relation to what is now called pyorrhea.

"Scurvy as we had it at Andersonville was a very different disease from pyorrhea, and I don't think it is any kin to it.

"Persons taking this disease always became languid and began to break out on the hands and wrists and all exposed parts of the body like sun burn. Their minds became deranged in by far the greater number of cases. Their bowels became deranged at the same time, and it then only took a few days to produce death. A great portion of the last days of summer and early fall it was nothing uncommon to have from ninety to one hundred and eight deaths in the twenty-four hours."

